# Transglutaminase 2 cross-linking activity is linked to invadopodia formation and cartilage breakdown in arthritis

**DOI:** 10.1186/ar3899

**Published:** 2012-07-04

**Authors:** Annie Lauzier, Martine Charbonneau, Marilène Paquette, Kelly Harper, Claire M Dubois

**Affiliations:** 1 Immunology Division, Department of Pediatrics, Faculty of Medicine and Health Sciences, Université de Sherbrooke, 3001 12th North Avenue, Sherbrooke, Quebec, J1H 5N4, Canada

## Abstract

**Introduction:**

The microenvironment surrounding inflamed synovium leads to the activation of fibroblast-like synoviocytes (FLSs), which are important contributors to cartilage destruction in rheumatoid arthritic (RA) joints. Transglutaminase 2 (TG2), an enzyme involved in extracellular matrix (ECM) cross-linking and remodeling, is activated by inflammatory signals. This study was undertaken to assess the potential contribution of TG2 to FLS-induced cartilage degradation.

**Methods:**

Transglutaminase (TGase) activity and collagen degradation were assessed with the immunohistochemistry of control, collagen-induced arthritic (CIA) or TG2 knockdown (shRNA)-treated joint tissues. TGase activity in control (C-FLS) and arthritic (A-FLS) rat FLSs was measured by *in situ *5-(biotinamido)-pentylamine incorporation. Invadopodia formation and functions were measured in rat FLSs and cells from normal (control; C-FLS) and RA patients (RA-FLS) by *in situ *ECM degradation. Immunoblotting, enzyme-linked immunosorbent assay (ELISA), and p3TP-Lux reporter assays were used to assess transforming growth factor-β (TGF-β) production and activation.

**Results:**

TG2 and TGase activity were associated with cartilage degradation in CIA joints. In contrast, TGase activity and cartilage degradation were reduced in joints by TG2 knockdown. A-FLSs displayed higher TGase activity and TG2 expression in ECM than did C-FLSs. TG2 knockdown or TGase inhibition resulted in reduced invadopodia formation in rat and human arthritic FLSs. In contrast, increased invadopodia formation was noted in response to TGase activity induced by TGF-β, dithiothreitol (DTT), or TG2 overexpression. TG2-induced increases in invadopodia formation were blocked by TGF-β neutralization or inhibition of TGF-βR1.

**Conclusions:**

TG2, through its TGase activity, is required for ECM degradation in arthritic FLS and CIA joints. Our findings provide a potential target to prevent cartilage degradation in RA.

## Introduction

Rheumatoid arthritis is a disabling autoimmune disease characterized by a chronic state of inflammation that can affect several organs and tissues, although small joints of patients are most commonly affected. The increased inflammatory cell infiltration of the synovium is accompanied by a modification of the resident synovial cell population. Activation of fibroblast-like synoviocytes (FLSs) leads to the release of a broad array of mediators that act on cells of the immune system as well as resident joint cells, exacerbating the inflammatory response and resulting in articular cartilage and bone damage [1].

Many factors present in the inflamed microenvironment of the joints have been shown to activate FLSs. These include various cytokines and chemokines [2-4], growth factors [5], extracellular matrix fragments [6,7], and hypoxia [8]. The selective pressure from the inflammatory environment generates intrinsic changes in RA-FLSs leading to an enhanced ability to attach to cartilage, invade through matrix, and synthesize degradative enzymes [9]. We recently reported that the ability of A-FLSs to degrade ECM is dependent on the formation of specialized structures resembling invadopodia in tumor cells, which are invasive structures involved in basement membrane degradation, or podosomes, the bone-resorbing structures found in osteoclasts [10,11]. Characterization of the A-FLS structures indicated that they contained actin components, activated kinases (Scr), and the metalloproteinases MMP3 and MMP-13, which are known to be particularly efficient at inducing cartilage degradation. They were found in cells at the cartilage/pannus junction, well positioned for cartilage degradation. Importantly, interference with the formation of invadopodia in A-FLSs by Src kinase inhibition impeded ECM degradation *in vitro *and cartilage degradation in a model of collagen-induced arthritis (CIA) [11], strongly suggesting that invadopodia are physiological structures involved in cartilage destruction.

Transglutaminase 2 (TG2) is a multifunctional enzyme that has been associated with wound healing and inflammatory diseases (for review, see [12]). TG2 is ubiquitous and generally located in the cytosol in a catalytically inactive form because of low intracellular calcium and high GTP concentrations. It can also be found in the nucleus, the inner surface of the plasma membrane, or can be secreted, in which case, it localizes in the ECM or at the cell surface [13]. TG2 is known mostly for its ability to catalyze the cross-linking of proteins by transamidation of a glutamine residue to a lysine residue, a reaction that requires Ca^2+ ^[14]. The resulting ε-(γ-glutamyl)-lysine bond is resistant to proteases and confers increased stability to protein complexes involved in cellular functions, including apoptosis and matrix remodeling. Besides its transglutaminase (TGase) activity, TG2 can act as a protein disulfide isomerase and can display protein kinase, GTPase, and DNA nuclease activities [15-17]. TG2 can also serve as an adaptor to facilitate cell adhesion to fibronectin by interacting with β-integrins, syndecan, or cell-adhesion receptors [18-20]. The expression of TG2 is regulated by cytokines and growth factors involved in inflammation, and TG2 was found to be overexpressed in the RA synovium [12]. Transforming growth factor-β (TGF-β) induces TG2 expression through its effect on a response element located in the promoter region of the gene [21]. TG2, in turn, can upregulate the expression of TGF-β through nuclear factor (NF)-κB activation, which is concomitant with an increase in bioactive TGF-β in the extracellular environment [22], thus enforcing a positive-feedback loop. TG2 expression is also increased by TNF-α through NF-κB activation in liver cells [23] and by interleukin-1 (IL-1) in cartilage tissues [24]. TG2 overexpression is linked to increased aggressivity in many cancer cells [25], whereas TG2-knockout mice show reduced cartilage degradation in a model of osteoarthritis [26].

TG2 and the degradation capacity of activated FLSs are both stimulated as the result of the inflammatory environment present in the arthritic synovium. We therefore investigated the impact of the modulation of TG2 levels and/or activity on cartilage degradation in CIA rats and in invadopodia formation in FLSs. We showed that the TGase activity of TG2 was associated with collagen type II degradation in CIA joints. Of significance, knockdown of TG2 resulted in a strong reduction in the degradation of collagen. We further demonstrated, for the first time, that the regulation of invadopodia formation was related to the TGase activity of TG2, and that this effect was dependent on TGF-β signaling. Taken together, our findings provide evidence of the existence of a new TG2-dependent pathway that influences FLS-mediated degradation in arthritic joints.

## Materials and methods

### Induction and clinical evaluation of arthritis

All experimental procedures involving animals were conducted under protocols approved by the Ethics Committee on Animal Research of the University of Sherbrooke, in accordance with regulations of the Canadian Council on Animal Care. Female Lewis/SsNHsd rats (100 to 124 g) were purchased from Charles River Laboratories (St-Constant, QC, Canada). Arthritis was induced by intradermal injection of type II collagen at the base of the tail, as described [11]. In the case of *in vivo *TG2 inhibition, rats received one intraarticular injection of 3 × 10^9 ^units of a lentivirus harboring TG2-shRNA or control (scrambled) shRNA, 10 days after CIA induction.

### Histology

Tissue sections from the left hind-knee joints were processed immediately after excision by following a standardized paraffin-embedding protocol. Tissue sections were rehydrated, treated with trypsin 1%, and immunohistochemical staining was performed according to the standard avidin-biotin immunoperoxidase complex technique by using a mouse monoclonal ε-(γ-glutamyl)-lysine bond antibody (Abcam (Cambridge, MA, USA) ab424; 1:100), a rabbit polyclonal collagenase cleaved type I and II collagen (Col 2 3/4 short) antibody (Ibex Pharmaceutical (Montreal, QC, Canada) Ab 50-1035; 1:75), or a mouse monoclonal TG2 (Thermo Scientific (Rockford, IL, USA) TG100; 1:50). DAB, SG, or VIP (Vector Laboratories Inc., Burlington, ON, Canada) was used for the detection of labeled proteins. Some sections were counterstained with Harris hematoxylin. Tissue sections from rats in each group were treated simultaneously to palliate interexperimental staining variations.

### Modified Mankin grading

Sections were stained by using hematoxylin/eosin and the safranin O/Fast green staining protocols to allow visualization of cartilage structure. Pathology scores were evaluated for each tissue section by three blinded observers using a modified Mankin scoring system, as described [27]. In the cartilage structure subcategory of the scoring system, the extent of cartilage invasive zones was evaluated instead of cartilage clefts, the better to account for the cartilage changes observed in CIA.

### Analysis of immunolabeled specimens

Analysis of joint section (synovial membrane and cartilage) was performed by using an Axioskop 2 phase-contrast/epifluorescence microscope (Carl Zeiss, Inc., Toronto, ON, Canada) equipped with a 10× or 40× objective at the same level of incident illumination for each slide in the experiment. The condenser was adjusted to bright-field transmission light microscopy. Photomicrographs of six (for 40× magnification) or three (for 10× magnification) random areas within the synovial membrane or cartilage were captured with Retiga SRV cooled color digital camera (Qimaging, Surrey, BC, Canada). The intensity of labeling in the synovial membrane or a representative portion of cartilage in each image was analyzed by using the IHC quantification technique validated by Pham *et al. *[28]. Images were converted to CMYK in the FIJI software (Open Source). Next, the gray image of the yellow (Y) channel was extracted, and chromogen intensities were analyzed by using the Image Pro software (Media Cybernetics Inc., Bethesda, MD, USA). Results are expressed as the sum of labeling intensity (density) relative to the total area, from two different tissue sections for each rat.

### Cell cultures

Synovial membranes were removed from the right hind-knee joints of experimental rats, and cells were isolated by collagenase type IV digestion, as described [29]. Four human cell lines isolated from patients diagnosed with RA and undergoing arthroplasty (RA-FLS) or three cell lines isolated from control joints with no evidence of disease (C-FLS) were obtained from Asterand (Detroit, MI, USA). All synovial tissues were obtained with Institutional Review Board approval and appropriate informed consent. Cells were cultured in DMEM-F12 supplemented with 10% FBS and 40 μg/ml gentamycin. The cells were used between passages 3 and 8.

### Plasmids and transfections

pcDNA3.1-wtTG2 and pcDNA3.1-W241A-TG2 were generously provided by Dr. Gail V.W. Johnson (University of Rochester, Rochester, NY, USA). GFP-lentiviral shRNAs targeting rat TG2 and control (scrambled) shRNA plasmids were from ezBiolab (Carmel, IN, USA). Control and mouse/rat FXIIIA targeting pLKO.1-shRNA plasmids were from Thermo Scientific. Viral particles were generated by transient transfection of 293T cells with the ViraPower lentiviral expression system (Invitrogen, Burlington, ON, Canada). Synoviocytes were tested in the different assays 24 hours after transient transfection by using Fugene 6 (Roche Diagnostics, Laval, QC, Canada) or 48 hours after lentivirus infection with Polybrene (5 μg/ml; Santa Cruz Biotechnology, Santa Cruz, CA, USA). With this procedure, a high efficiency (about 60%) of lentivirus infection was achieved in both rat and human synoviocytes. Cells expressing FXIIIa-shRNAs were selected with a 72-hour puromycin treatment.

### Invadopodia assays

Coverslips were prepared by using Oregon Green^488^-conjugated gelatin (Invitrogen) at a final concentration of 1%, as described [30]. Thirty thousand cells were seeded on each coverslip and incubated for 40 hours. Cells were fixed with 1% paraformaldehyde and stained with DAPI and Alexa^647^-conjugated phalloidin (Invitrogen). Cells were visualized with fluorescence microscopy. Invadopodia were identified by actin-rich areas of matrix degradation characterized by loss of green fluorescence. Three hundred cells were counted per coverslip. In the case of TG2-overexpressing cells, TG2 was labeled by using anti-TG2 antibodies (Thermo Scientific CUB7402), and invadopodia formation was counted for TG2-transfected cells only. For inhibition and activation assays, dithiothreitol (DTT; Sigma, Oakville, ON, Canada), Cystamine (Sigma), KCC-009 (provided by Dr K.M. Rich, Washington University, St. Louis, MO, USA), Z-DON (Zedira, Darmstadt, Germany), TGF-β (Peprotech, Rocky Hill, NJ, USA), LY364947 (Tocris Bioscience, Bristol, UK), or TGF-β-neutralizing antibody (R&D Systems (Minneapolis, MN, USA) Ab100-NA; 15 μg/ml) was added 30 minutes after cell plating. In the case of invadopodia assays of GFP-shRNA-expressing cells, Texas-Red-conjugated 1% gelatin slides were prepared as described [31], and the number of invadopodia (loss of red fluorescence) was counted for GFP-positive cells. To quantify the areas of degradation, pictures of fluorescent matrix were analyzed by using ImagePro software, and degradation areas associated with positive cells were calculated in pixels, for a minimum of 25 cells per coverslip.

### Immunofluorescence and confocal microscopy

Synoviocytes cultured on coverslips for 4 to 6 hours were prepared as described [11] and stained by using antibodies against p-cortactin (Cell Signaling (Danvers, MA, USA) Ab 45569; 1:75), TG2 (ThermoScientific CUB 7402; 1:50), and stained for actin by using Texas Red- or Alexa^647^-conjugated phalloidin (Invitrogen; 1:200 or 1:50). Negative control slides were treated with isotype-matched primary antibodies, followed by secondary antibodies. Confocal images were acquired by using a Fluoview 1000 scanning confocal microscope (Olympus, Richmond Hill, ON, Canada) in-line with an inverted microscope equipped with a 60× oil-immersion objective. Color channels were scanned sequentially to avoid overlapping signals.

### TGase activity assay

To evaluate the activity level of TG *in vivo *and *in vitro*, EZ-link pentylamine-biotin (PAB) (Pierce/Thermo Scientific) was used as an exogenous TGase substrate. For the live cell activity assay, 30,000 cells were allowed to adhere to 0.2% gelatin-coated coverslips for 30 minutes and incubated for 3 hours with 0.75 m*M *PAB after addition of treatments, as specified in the figure legends. Coverslips were fixed with 1% PFA, permeabilized with 0.05% saponin, and blocked with 2% BSA. Cross-linked biotin was detected with Alexa^546^-conjugated streptavidin (Invitrogen). Mean fluorescence intensity per cell was calculated from photographs of 20 randomly selected cells for each treatment. To assess TGase activity in articular homogenates, tissue lysates were prepared from freshly excised control and CIA rats (21 days after induction) synovial membranes, homogenized by using a bead mill (Retsch, Newtown, PA, USA) in TGase reaction buffer (50 m*M *Tris, pH 7.5; 150 m*M *NaCl; 5 m*M *CaCl_2_; Complete (Roche)). Lysates were centrifuged, and the protein content of the supernatants was determined by using the Bradford method (Bio-Rad Laboratories, Mississauga, ON, Canada). Equal amounts of protein were supplemented with 1 m*M *DTT and inhibitor (Z-DON, 100 μ*M*; KCC-009, 250 μ*M*) or vehicle. TGase enzymatic activity was started by addition of 5 m*M *PAB and allowed to proceed at 37°C for 4 or 20 hours. Reaction was stopped by addition of SDS loading buffer and sample boiling. Samples were separated with SDS-PAGE and transferred onto nitrocellulose membranes. To assess TGase activity in articular joints, rat hind knees were injected with 0.5 m*M *PAB in PBS, 1.2 m*M *CaCl_2_, 0.5 m*M *MgCl_2_, with or without 10 m*M *Z-DON or 250 *M *KCC-009, and incubated at 37°C for 4 hours. Tissues were processed as described earlier. In both experiments, biotin-labeled proteins were detected with streptavidin-conjugated alkaline phosphatase.

### Western blot analysis

Whole-cell extracts were prepared by lysis of overnight serum-starved cells in RIPA buffer. ECM protein extracts were prepared from cells grown for 72 hours in complete medium, as described [22]. In brief, cells were detached with 2 m*M *EDTA/PBS. The remaining cell-assembled matrix was washed with 2 m*M *EDTA/PBS/0.1% deoxycholate and scraped in 2× strength Laemmli gel-loading buffer to obtain the ECM fraction. Proteins were immunoblotted as described [29] by using anti-TG2 (ThermoScientific TG100; 1:500), anti-LAP (R&D Systems Ab-246-NA; 1:1,000) or anti-actin (Sigma Ab A5060; 1:5,000) antibodies. Band intensities were analyzed by using the Quantity One software (Bio-Rad Laboratories).

### TGF-β ELISA and luciferase assay

Quantikine ELISA for Human TGF-β 1 (R&D Systems) was used, following the manufacturer's instructions, with acid-activated A-FLS supernatants. For luciferase assays, cells were transiently transfected with p3TP-Lux [32] by using 1 mg/ml PEI (Polysciences, Warrington, PA, USA) in DMEM-F12 supplemented with KnockOut Serum Replacement (Invitrogen). Twenty-four hours after transfection, cells were starved and treated overnight, as indicated in the figure legends. Cell lysates were assayed for luciferase activity, as described [29].

### Statistical analysis

The paired or unpaired Student *t *test or one-way ANOVA test was used to assess statistical significance. A *P *value smaller than 0.05 was considered significant.

## Results

### TG2 and TGase activity are associated with degradation of type II collagen in CIA joints

**Figure 1:**
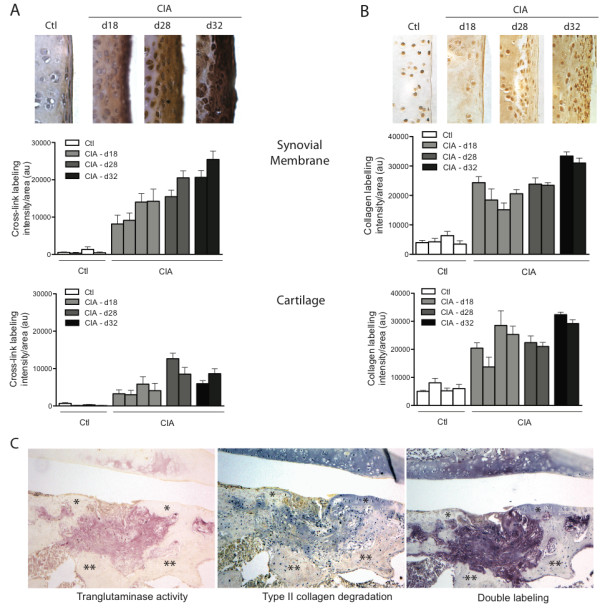
**The formation of ε-(γ-glutamyl)-lysine bonds is associated with collagen degradation in CIA**. Images (40× magnification) of representative zones of synovial membrane/cartilage from articular tissue sections of control and arthritic rats at different times (18, 28, and 32 days) after immunization with type II collagen. Tissues were immunostained with **(A) **ε-(γ-glutamyl)-lysine bond antibody and hematoxylin or **(B) **degraded collagen antibody. Associated graphs show relative labeling intensity calculated from six random fields for each tissue section in the synovial membrane (upper graphs) or in the cartilage (lower graphs). Each column represents mean value ± SEM of total fields from two different tissue sections per individual rat (Ctl, *n *= 4; CIA, *n *= 8). **(C) **Representative images (10× magnification) of immunostaining of ε-(γ-glutamyl)-lysine bonds (red), degraded collagen (blue), and double labeling (purple) in successive CIA tissue sections (*cartilage; **bone).

Knee joints of control and CIA rats were prepared for immunohistochemistry (IHC) and labeled with an ε-(γ-glutamyl)-lysine bond-specific monoclonal antibody to obtain an estimation of the cross-linking activity of TGase or TG2-specific antibodies. As revealed by the intensity of labeling, both TG2 and cross-linking activity were detectable at day 18 after the induction of arthritis and increased progressively in the synovial membrane and adjacent cartilage of CIA rats, except at day 32, when the intensity of TG2 labeling declined (Figure 1A and Additional file 1, Figure S1). At days 18 to 32, TG2 and cross-linking labeling were predominantly expressed in the hyperplastic synovial lining and at the synovial lining/cartilage junction, with less activity found within the chondrocyte layer of the cartilage (Figure 1A and Additional file 1, Figure S1). To define whether TG2 is linked to the cross-linking activity found in synovial membranes, we assessed *in vivo *TGase activity by measuring biotin-pentylamide (BAP) incorporation in homogenates of normal and arthritic synovial membrane or in whole articular joints in the presence or absence of the inhibitors KCC-009, an irreversible TGase inhibitor [33], or Z-DON, a selective TG2 inhibitor [34]. The results indicated that BAP incorporation into proteins from arthritic synovium homogenates was increased (as revealed by the intensity of the bands) compared with normal rat synovium homogenates. In addition, the bands from both normal and arthritic homogenates incubated in the presence of the TG2-specific inhibitor Z-DON showed reduced intensity compared with vehicle, whereas a stronger reduction was obtained by using the TGs inhibitor KCC-009 (see Additional file 2, Figure S2A). Furthermore, direct assessment of TGase activity into articular joints (through intraarticular (IA) BAP injection) indicated that the TGase activity inhibited by Z-DON was located mainly in the synovial lining of arthritic joints (see Additional file 2, Figure S2B). Similar results were obtained with the KCC-009 inhibitor (data not shown).

To evaluate the extent of cartilage damage, the articular tissues were immunostained by using an antibody that detects a newly exposed epitope within collagenase-cleaved type II collagen [35]. Results showed that the intensity of degraded collagen staining in both synovial membrane and underlying cartilage increased with time during the development of arthritis (Figure 1B). In sections stained with hematoxylin/eosin and safranin O, evaluation of histopathologic changes, according to a modified Mankin scoring system [27], revealed a loss of structural integrity of the cartilage, a reduction in proteoglycan content, and synovial membrane hyperplasia, which were more pronounced as arthritis progressed (see Additional file 3, Figure S3). The obtained histologic scores closely reflected changes observed with TG2, TGase, and collagen-degradation assessments. In addition, double labeling of joint tissues revealed robust TGase activity that colocalized with collagen degradation at sites of synovial membrane invasion (Figure 1C). These results clearly suggested an association between cartilage degradation and TGase activity in CIA joints.

### Knockdown of TG2 reduces cartilage degradation in CIA

**Figure 2:**
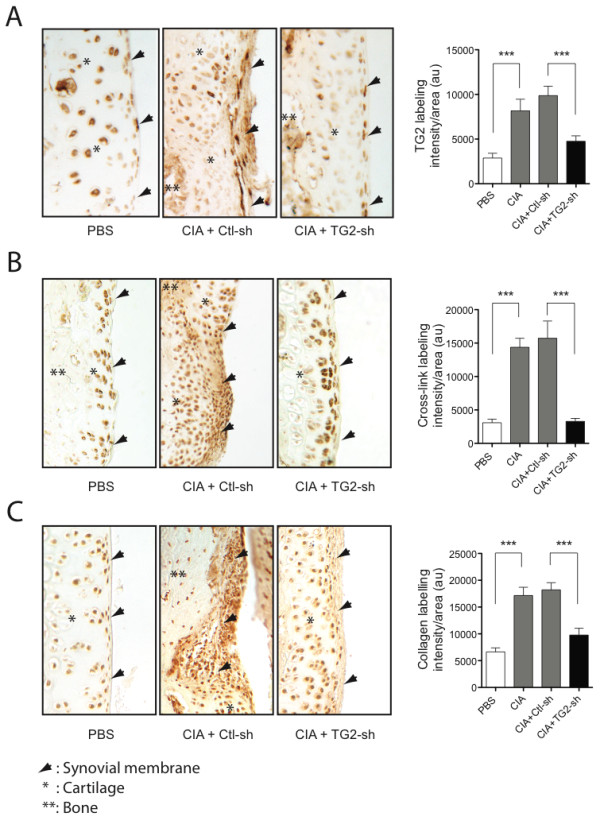
**TG2 knockdown reduces collagen degradation in CIA rats**. Representative images (10× magnification) of immunostaining of **(A) **TG2, **(B) **ε-(γ-glutamyl)-lysine bonds, and **(C) **degraded collagen in the synovial membranes from control rats (PBS; *n *= 5), CIA rats injected with control (CIA+Ctl-sh; *n *= 6), or TG2 (CIA+TG2-sh; *n *= 5) shRNA-expressing lentivirus. Associated graphs show relative labeling intensities for these experimental groups and CIA control (*n *= 4) rats, calculated from three random fields for each tissue section. Values are expressed as the mean ± SEM (***P < 0.001).

We next examined whether silencing the expression of TG2, the main active transglutaminase in articular cartilage [36], affected cartilage degradation. For this, TG2-shRNA- or control-shRNA-expressing lentivirus was injected IA into CIA rats. Injections were performed at day 10 to avoid interference with the initiation of the immune response. Tissue sections from rat hind paws were immunostained with an antibody directed against TG2 or ε-(γ-glutamyl)-lysine epitope to assess efficiency of the treatment. Results indicated that the high levels of TG2 labeling and TGase activity observed in arthritic synovial membranes from control-shRNA-treated rats were markedly reduced in TG2-shRNA-treated animals, reaching levels comparable to control (nonarthritic) joints (Figure 2A, B). In parallel, immunostaining by using an antibody directed against fragments of type II collagen showed that the extensive zones of collagen degradation observed in the joints of CIA rats were similarly reduced in tissues of CIA rats treated with TG2-shRNA (Figure 2C). Collectively, these data suggested that TG2 is the main enzyme involved in cross-linking activity and cartilage degradation in CIA rats.

### TG2 expression and TGase activity are increased in arthritic FLSs

**Figure 3:**
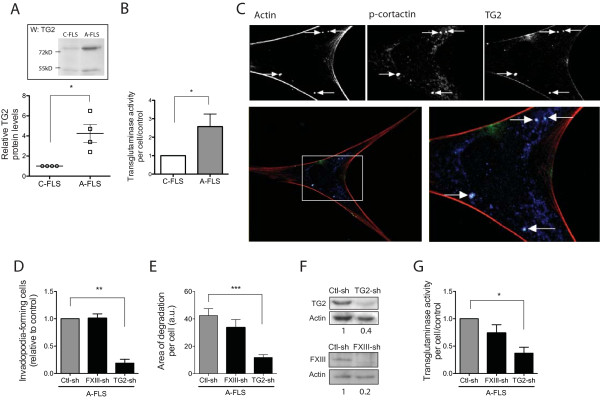
**TG2 expression and matrix degradation are increased in FLSs from CIA rats**. **(A) **Control FLSs (C-FLSs) and arthritic FLSs (A-FLSs) were cultured for 72 hours, and TG2 in the ECM laid down by the cells was visualized with immunoblotting. Graph shows the densitometric analysis of TG2/actin ratio (*n *= 4) with representative immunoblot (insert). **(B) **C-FLSs and A-FLSs were cultured on gelatin, and TGase activity was measured by using *in situ *5-(biotinamido)-pentylamine incorporation assay. Graph shows mean labeling intensities of 20 cells per experiment (*n *= 3). **(C) **Representative confocal microscopy image (60×) of the basal surface of the cell showing localization of endogenous F-actin (red), p-cortactin (blue), and TG2 (green) and overlay of the three channels (merge) in RA-FLSs grown on gelatin for 16 hours. Arrows show the position of the actin and p-cortactin dots that colocalize with TG2. **(D **through **G) **A-FLSs were transfected with FXIIIa-, TG2-, or their respective control (scrambled)-shRNA-expressing lentivirus. (D) The percentage of invadopodia-forming cells was counted for 100 GFP-expressing cells (TG2-shRNA) or 300 cells (FXIIIa-shRNA), and (E) the mean area of degradation per cell was calculated for 25 transfected cells (*n *= 3). (F) Representative immunoblot of TG2 or FXIIIa and actin showing knockdown with respective shRNA. (G) *In situ *TGase activity was measured in a minimum of 20 cells (*n *= 3). Values are expressed as the mean ± SEM (*P < 0.05; **P < 0.01; ***P < 0.001).

Because it is well established that FLSs play a major role in cartilage degradation, we next assessed the levels of TG2 expression and TGase activity in rat synovial cell cultures. Immunoblot analysis of intracellular, secreted, and matrix-bound TG2 revealed an absence of consistent differences with respect to intracellular and secreted TG2 between experimental and control rats (data not shown). In marked contrast, a reproducible increase in the deposition of TG2 in the laid-down ECM was found in arthritic FLS cultures compared with control FLSs (Figure 3A). In addition, A-FLSs cultured on a gelatin matrix displayed consistently higher TGase activity (averaging 2.5-fold) than did C-FLSs (Figure 3B and Additional file 4, Figure S4A).

### Knockdown of TG2 reduces the formation of invadopodia by arthritic FLSs

Recent evidence indicated that the ECM degradation activity of rat A-FLS *in vitro *and in arthritic joints is associated with the formation of ECM-degrading cell structures, which are reminiscent of invadopodia produced by cancer cells or podosomes in osteoclasts [10,11]. We therefore investigated whether the modulation of TG2 in A-FLSs affected their capacity to form ECM-degrading invadopodia. Confocal microscopy analysis revealed that 81% ± 4.3% of invadopodia structures, identified by clusters of actin and p-cortactin, two *bona fide *markers of invadopodia [37], colocalized with TG2 (Figure 3C). Of significance, knockdown of TG2 caused a drastic decrease in the percentage of cells forming invadopodia, as well as in the extent of ECM degradation induced by invadopodia (Figure 3D, E). In contrast, knockdown of FXIIIa, a parental TG, which is also expressed in synovial cells (Figure 3F), failed to reduce invadopodia formation or ECM degradation (Figure 3D, E). Immunoblotting experiments confirmed the efficiency of TG2 and FXIIIa knockdown (Figure 3F). Furthermore, TG2 knockdown resulted in a strong decrease in total TGase activity, whereas knockdown of FXIIIa had a milder effect (Figure 3G and Additional file 4, Figure S4B), confirming that TG2 was responsible for the majority of the TGase activity in A-FLSs. Together, these results indicated that TG2 was involved in the formation of ECM-degrading invadopodia in arthritic FLSs.

### The TGase activity of TG2 is involved in invadopodia formation in rat and human FLSs

**Figure 4:**
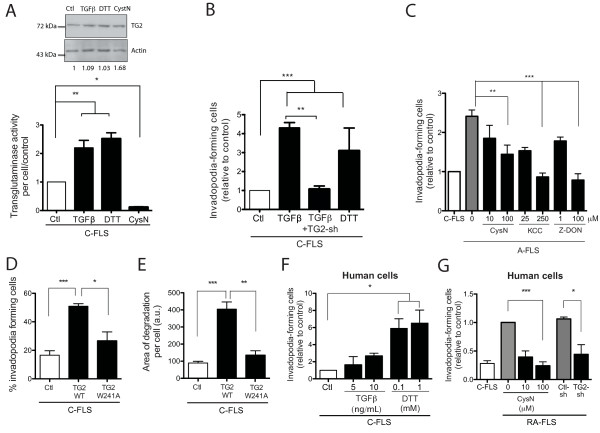
**TGase activity of TG2 is involved in *in vitro *matrix degradation by FLSs**. **(A) **C-FLSs were incubated with TGF-β (20 ng/ml), DTT (1 m*M*), or cystamine (100 μ*M *). After 3 hours, TG2 expression was evaluated with Western blotting (*n *= 2), and TGase activity was measured for cells incubated on gelatin matrix (*n *= 7). **(B) **C-FLSs or C-FLSs transfected with TG2-shRNA-expressing lentivirus or **(C) **A-FLSs were cultured on a gelatin matrix, incubated with TGF-β (20 ng/ml), DTT (1 m*M*), cystamine (10 or 100 μ*M*), KCC-009 (25 or 250 μ*M*), or Z-DON (1 or 100 μ*M*), and the percentage of cells forming invadopodia at 24 hours was counted for 300 cells per experiment (*n *= 3). **(D, E) **C-FLSs were transfected with wild-type TG2 (TG2 WT), TG2-W241A, or empty vector and cultured on gelatin for 24 hours. (D) The percentage of cells forming invadopodia for 100 transfected cells (overexpressing TG2) per experiment (*n *= 3) and (E) the mean area of degradation per cell calculated for 25 transfected cells (*n *= 3) is shown. **(F) **Human C-FLSs treated with TGF-β (5 and 10 ng/ml) or DTT (0.1 and 1 m*M*) or **(G) **synoviocytes from patients with rheumatoid arthritis (RA-FLSs) transfected with GFP-tagged control- or TG2-shRNA-expressing lentivirus or treated with cystamine (10 and 100 μ*M*) were grown on gelatin, and the percentage of GFP-expressing invadopodia-forming cells was counted at 24 hours (*n *= 3). Values are expressed as the mean ± SEM (*P < 0.05; **P < 0.01; ***P < 0.001).

We next examined whether modulation of the TGase activity of TG2 affected the capacity of FLSs to form invadopodia. For this, TGF-β, a potent inducer of TG2 and its TGase activity [21,38], and DTT, a disulfide-bond reducer that unfolds TG2, inducing its TGase activity [39], were added to C-FLS at the onset of invadopodia assays. Results showed that each treatment induced a strong increase in cross-linking activity (2.2- and 2.5-fold increases, respectively) as compared with untreated C-FLS, whereas the relative levels of TG2 protein were only mildly affected (Figure 4A and Additional file 4, Figure S4C). In contrast, cystamine, a competitive inhibitor of TGase [40], strongly reduced the cross-linking activity of C-FLS (Figure 4A and Additional file 4, Figure S4C). These TGase inhibitors/activators were also tested in invadopodia assays. TGF-β and DTT treatment induced 4.3- and 3.1-fold increases, respectively, in the percentage of control synovial cells forming invadopodia (Figure 4B). The stimulatory effect of TGF-β was blocked by shRNA-dependent TG2 depletion (Figure 4B). In addition, preincubation of A-FLSs with cystamine, the TGase inhibitor KCC-009, or the TG2-specific inhibitor Z-DON, induced a significant dose-dependent decrease in the formation of invadopodia without affecting cell viability (Figure 4C and data not shown). These results indicated that modulation of the TGase activity of TG2 influenced invadopodia formation in FLSs. Further to evaluate the role of the TGase activity of TG2, C-FLSs were transfected with a TGase-defective mutant (TG2-W214A), which is efficiently secreted (data not shown), or wild-type TG2 (TG2 WT), and assayed for invadopodia formation. Overexpression of TG2-W214A resulted in weak invadopodia formation. Furthermore, this mutant TG2 failed to promote ECM degradation, in contrast to control cells harboring WT TG2 (Figure 4D, E). These results strongly suggest that TG2 acted through TGase activity for the regulation of invadopodia formation in rat FLSs.

To determine whether our findings could be applied to human arthritis, studies were performed by using synovial cells derived from control (arthritis-free; C-FLSs) and rheumatoid arthritis patients (RA-FLSs). Treatment of C-FLSs with either TGF-β or DTT caused 2.7- and 6.5-fold increases, respectively, in invadopodia formation (Figure 4F). In contrast, silencing TG2 expression by using TG2-shRNA or inhibition of TGase activity with cystamine in synovial cells from RA patients resulted in marked decreases in invadopodia formation, reaching levels similar to those found in control FLS (Figure 4G). These data led us to conclude that TG2 acted, at least in part, through its TGase activity to regulate invadopodia formation in rat and human synovial cells.

### TGase-mediated induction of matrix degradation is dependent on TGF-β production and signaling in FLSs

**Figure 5:**
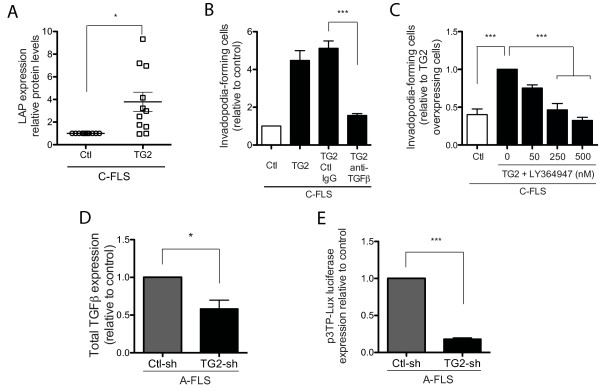
**TGase-mediated induction of matrix degradation is modulated by TGF-β in FLSs**. **(A-C) **C-FLSs were transfected with TG2 or empty vector. (A) Immunoblot analysis using anti-LAP antibody in cell lysates. Densitometric measurements of LAP-to-actin ratios are shown (*n *= 11). (B) Cells were deposited on a gelatin matrix and incubated with control (Ctl)-IgG or anti-TGF-β antibodies. After 24 hours, the percentage of invadopodia-forming cells was counted (*n *= 4). (C) Cells were deposited on a gelatin matrix and incubated for 24 hours with LY-364947 (50 n*M *to 500 n*M*), and the percentage of invadopodia-forming cells was counted (*n *= 4). **(D, E) **Arthritic FLSs were transfected with control- or TG2-shRNA-expressing lentivirus. (D) Total TGF-β was measured with ELISA (*n *= 4). (E) shRNA-expressing cells were transiently transfected with p3TP-LuX, and luciferase assays were performed (*n *= 4). Values are expressed as the mean ± SEM (*P < 0.05; ***P < 0.001).

To gain insight into the mechanism by which TG2 influences invadopodia formation and ECM degradation, we investigated the ability of TG2 to affect the bioavailability of TGF-β. TG2 has been shown to enhance TGF-β production and to contribute to TGF-β bioactivation by favoring the binding of latent TGF-β-binding protein-1 to ECM, which is a prerequisite to TGF-β release [22,41]. Overexpression of TG2 in C-FLSs resulted in a 4.3-fold increase in the production of TGF-β (Figure 5A), whereas a 4.6-fold increase in invadopodia formation was observed (Figure 5B) as compared with control cells transfected with an empty vector. The invadopodia-promoting effect of TG2 was completely inhibited by TGF-β1-3 neutralizing antibodies (Figure 5B). A similar inhibition was observed by treating TG2-overexpressing cells with the TGF-β R1 inhibitor, LY364947 (Figure 5C). In addition, silencing TG2 expression in arthritic FLSs inhibited TGF-β production (Figure 5D) and reduced TGF-β activity, as measured by using the p3TP-LuX reporter construct (Figure 5E). Taken together, these results indicate that endogenous TGF-β was part of the mechanism by which TG2 influenced invadopodia formation in arthritic synoviocytes. They further suggest that TGF-β expression and activity were regulated by TG2 in these cells.

## Discussion

An increase in the expression of TG2 or its transglutaminase activity is characteristic of numerous diseases [12]. TG2 is important in the regulation of cell/ECM interactions and is essential for many invasion-related cellular processes, including cell adhesion, cell spreading, and escape from anoikis, but the involvement of TGase activity in ECM degradation remains unknown. In this study, we present clear evidence that the TGase activity of TG2 is strongly associated with cartilage degradation in CIA. We also described, for the first time, a role for TG2 in invadopodia formation and ECM degradation by synovial cells from CIA rats and human arthritis patients.

The TGs family comprises at least eight members, including the tissue/cytosolic (TG1, TG2, TG3, TG4, TG5, TG6, and TG7) and the plasma (FXIIIa) form [42]. All of them seem to be widely distributed and to catalyze the formation of γ-glutamyl cross-links in a wide range of substrates that include histones, cytoskeleton proteins, collagens, osteopontin, osteonectin, and fibronectin [42]. Among the TGs, FXIIIa and TG2 were found to be expressed in low amounts in OA joints, whereas their level of expression was substantially increased (especially for TG2) in rheumatoid lesions [43]. Also, enhanced TGase activity has been detected in synovial fluids from RA patients [44]. Here, we showed that the level of TGase activity in CIA joints (expressed mostly by the hyperplastic/invading synovial membrane) is related to the extent of cartilage degradation. We also demonstrated that TG2 knockdown in arthritic joints caused more than 70% decrease in both TGase activity and cartilage degradation; such inhibition corresponds to the efficiency of lentivirus infection in synovial cells. Although arthritic synovial cells express both TG2 and FXIIIa, knockdown of the individual enzymes indicated that the majority of cross-linking activity in these cells arises from TG2. The capacity of these cells to form the ECM-degrading invadopodia structures was also associated with TG2. Although one cannot rule out the participation of other TGs, our data clearly suggested that TG2 is the major TGase involved in cartilage degradation in CIA. In support of our interpretation, Orlandi *et al. *[26] reported decreases in cartilage degradation in TG2-knockout mice, in a surgical model of osteoarthritis. Furthermore, exogenous administration of TG2 has been shown to enhance the severity of arthritis and joint destruction in mouse CIA [45].

FLSs are known to play a major role in the pathogenesis of arthritis through aggressive invasion and destruction of the underlying cartilage [46]. To obtain further insight into the mechanisms by which TG2 triggers cartilage degradation, we investigated the role of TG2 in the formation of invadopodia structures, which have been previously shown to be involved in joint destruction [11]. We found that the levels of TG2 deposited in the ECM were markedly increased in A-FLS cultures. By using modulators of TGase activity, TGase and TG2-specific inhibitors, or a TGase mutant of TG2, we showed that the degrading capacity of both rat and human A-FLSs as well as TG2-overexpressing normal FLS was, for the most part, related to its cross-linking (TGase) activity. The TGase activity of TG2 has been shown to be responsible for the increased deposition of matrix components such as fibronectin, which is potentially implicated in pannus formation [47], as well as the assembly of fibronectin in organized strands in the ECM [48]. This TG2-specific fibronectin remodeling has also been reported to increase cell adhesion to fibronectin [49], an important step in invadopodia formation by cancer cells. This suggests that matrix cross-linking by TG2 may influence invadopodia formation by altering the composition and conformation of important ECM constituents.

TG2 plays an important role in mesenchymal tissue remodeling, where its function in cell adhesion, mesenchymal transition, and matrix stabilization not only are important in normal wound healing but also can be involved in disease states such as cancer, tissue fibrosis, celiac disease, neurodegeneration, and osteoarthritis [22,42,50-52]. The ability of TG2 to regulate most of these various cellular and pathologic functions has been linked to its role in TGF-β expression and bioactivation in fibroblasts, breast and ovarian cancer cells, and renal epithelial cells [22,50,53-55]. Through its TGase activity, TG2 was found to regulate ECM deposition of the LTBP-complexed latent TGF-β [41,56], an important step in the regulation of TGFβ bioavailability. TG2 also modulated the expression and activation of TGF-β in an NF-κB-dependent manner [55,57]. We showed here that TG2 regulates TGF-β expression and TGF-β activity in synovial cells. Furthermore, inhibition of TGF-βR1 activation or TGF-β1-3 neutralization resulted in a significant loss in the formation of TG2-dependent invadopodia-like structures, suggesting that the modulation of endogenous TGF-β production/bioactivation is one mechanism by which TG2 influences invadopodia formation in A-FLS. Conversely, TG2 was found to be necessary for TGF-β-induced invadopodia formation, and our preliminary data indicate that this growth factor is in part responsible for enhanced TG2 secretion (needed for its extracellular cross-linking activity) in arthritic synovial (data not shown). These findings, together with the observation that TG2 has a canonic TGF-β responsive element within its promoter region [21,50], further suggest that enhanced TG2 expression/activation might be a way by which TGF-β propagates the amplification circuit in arthritic synovial cells.

Previous findings have indicated that exogenous TGF-β triggers invadopodia formation in breast cancer cells through a signaling mechanism involving Src activation [58]. Src activity has been found to be essential for the formation of invadopodia in a variety of cancer cells [59]. We also reported that invadopodia formation in A-FLS is related to Src activity [11]. Interestingly, inhibition of Src activity in synovial cells completely blunted invadopodia formation because of TG2 overexpression (see Additional file 5, Figure S5) and as documented here for TG2, Src activity was shown to be overexpressed in the synovial lining of CIA joints [11]. It is therefore possible that activated Src mediates the deleterious effect of TG2 on cartilage integrity. Further studies will be needed to elucidate the exact mechanism by which TG2 and Src influence invadopodia formation and cartilage degradation by FLS.

Although significant advances have been made in recent years in the treatment of arthritis, the commonly used approaches are associated with untoward side effects, and their efficacy against joint erosion remains limited [60]. Recent studies have highlighted the therapeutic potential of targeting TG2 for the treatment of various diseases, including neurodegenerative disorders, inflammatory diseases, and fibrotic diseases [53,61-65]. Our results indicate that TG2, mostly through its TGase activity, plays a critical role in experimental arthritis by promoting the breakdown of cartilage components by synovial cells. Inhibitors of the TGase activity of TG2 in rat models have shown efficacy in reversing the inflammation and fibrosis associated with chronic kidney diseases and inflammatory uveitis without apparent toxicity or side effects [66,67]. These observations suggest that TGase inhibition may provide a promising approach for the development of therapies aimed at stopping the process of cartilage degradation in arthritis.

## Abbreviations

A-FLS: arthritic FLS; C-FLS: control FLS; CIA: collagen-induced arthritis; ECM: extracellular matrix; FLS: fibroblast-like synoviocyte; LTBP: latent TGF-β-binding protein; PAB: pentylamine-biotin; RA-FLS: rheumatoid arthritis FLS; TG2: transglutaminase 2; TGase: transglutaminase; TGF-β: transforming growth factor beta.

## Competing interests

The authors declare that they have no competing interests.

## Authors' contributions

AL and MC carried out most of the experiments, participated in the design of the study, and drafted the manuscript. MP and KH helped in the *in vivo *animal assay, and KH helped to draft the manuscript. CMD conceived the study, participated in its design and coordination, and helped to draft the manuscript. All authors read and approved the final manuscript.

## Supplementary Material

Additional file 1 **Figure S1. TG2 expression is increased in synovial tissues in CIA**. **(A) **Representative images (40× magnification) of synovial membrane/cartilage from articular tissue sections of control and arthritic rats at different times (18, 28, and 32 days) after immunization with type II collagen. Tissues were immunostained with TG2. **(B, C) **Graphs show relative labeling intensity calculated from six random fields for each tissue section in the (B) synovial membrane or in the (C) cartilage. Each column represents mean value ± SEM of total fields from two different tissue sections per individual rat (Ctl, *n *= 4; CIA, *n *= 8). Click here for file

Additional file 2 **Figure S2. TG2 is linked to the cross-linking activity in synovial tissues**. **(A) **Synovial tissues from control (PBS) and CIA rats were incubated with biotin-pentylamide (PAB) in the presence or absence of 250 μ*M *KCC-009 (KCC) or 100 μ*M *Z-DON and revealed by Western blotting. **(B) **PAB was injected in PBS or CIA articulations with or without Z-DON (100 μ*M*) and revealed by immunochemistry with streptavidin-peroxidase. Neg Ctl, control tissue without biotin-pentylamide. Click here for file

Additional file 3 **Figure S3. Evaluation of CIA progression by using a modified Mankin scoring system**. Histopathologic classification of the severity of arthritic lesions was evaluated by using a modified Mankin scoring system. Graph shows mean values ± SEM for control and arthritic rats at different times (18, 28, and 32 days) after immunization with type II collagen (Ctl, *n *= 4; CIA, *n *= 8). Click here for file

Additional file 4 **Figure S4. Modulation of TGase activity is associated with TG2 regulation**. **(A-C) **TGase activity was measured by using *in situ *5-(biotinamido)-pentylamine incorporation assay in (A) C-FLSs or A-FLSs cultured on gelatin matrix. (B) A-FLSs were transfected with control- or TG2-shRNA-expressing lentivirus. (C) C-FLSs were incubated with TGF-β (20 ng/ml), DTT (1 m*M*), or cystamine (100 μ*Μ*). Click here for file

Additional file 5 **Figure S5. Src is implicated in TG2-mediated invadopodia formation**. C-FLSs were transfected with a TG2-expressing or a control (empty) plasmid and cultured on gelatin in the presence or absence of PP2. After 24 hours, the percentage of invadopodia-forming cells was counted. Values are expressed as the mean ± SEM (*P < 0.05; **P < 0.01; ***P < 0.001). Click here for file
