# Supplementary figures and images for: Transglutaminase 2 cross-linking activity is linked to invadopodia formation and cartilage breakdown in arthritis
Source: Arthritis Res Ther. 2012 Jul 4;14(4):R159. doi: 10.1186/ar3899 (PMC3580551; doi:10.1186/ar3899)

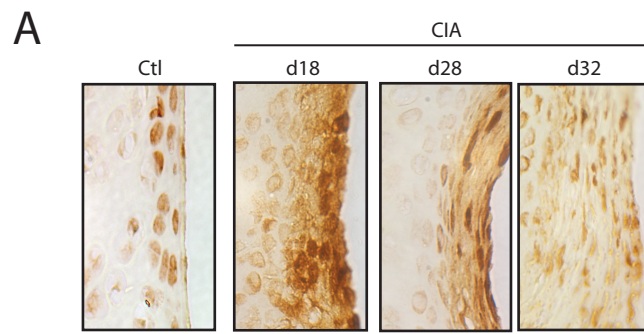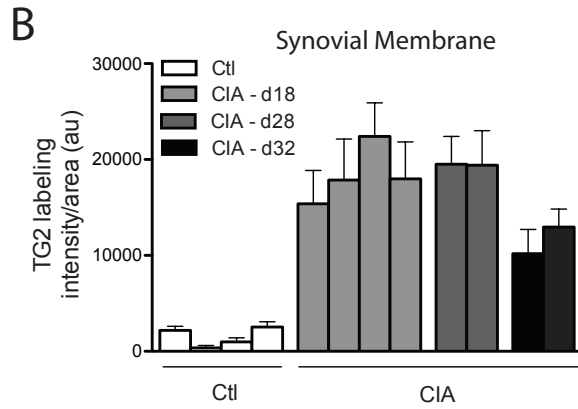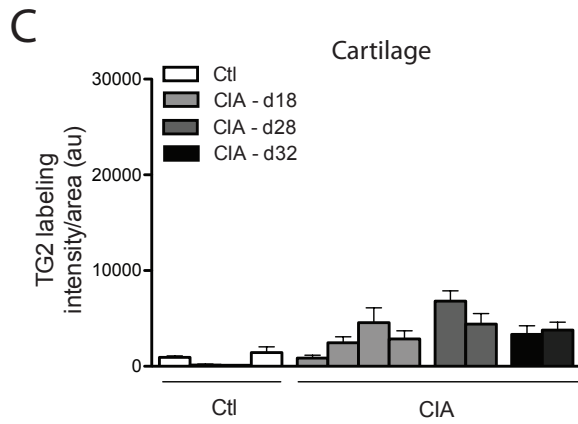

Supplement: Additional file 1 — Figure S1. TG2 expression is increased in synovial tissues in CIA. (A) Representative images (40× magnification) of synovial membrane/cartilage from articular tissue sections of control and arthritic rats at different times (18, 28, and 32 days) after immunization with type II collagen. Tissues were immunostained with TG2. (B, C) Graphs show relative labeling intensity calculated from six random fields for each tissue section in the (B) synovial membrane or in the (C) cartilage. Each column represents mean value ± SEM of total fields from two different tissue sections per individual rat (Ctl, n = 4; CIA, n = 8). [file ar3899-S1.PDF]

A

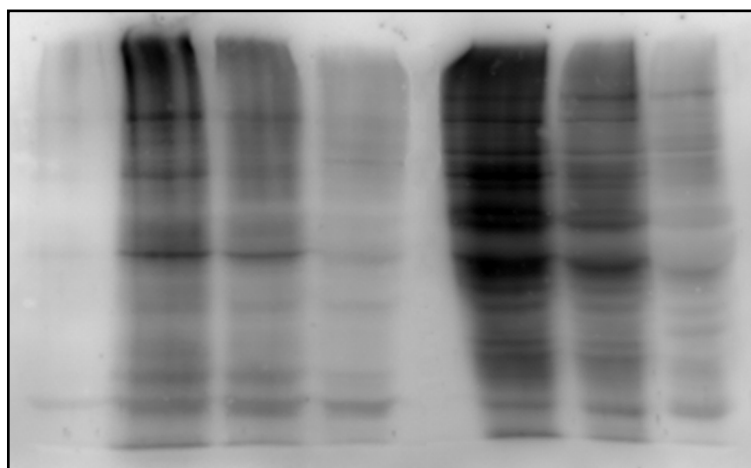

|            |          |       |     |  |          |       |     |
|------------|----------|-------|-----|--|----------|-------|-----|
| Neg<br>Ctl | Ctl      | Z-DON | KCC |  | Ctl      | Z-DON | KCC |
|            | PBS rats |       |     |  | CIA rats |       |     |

B

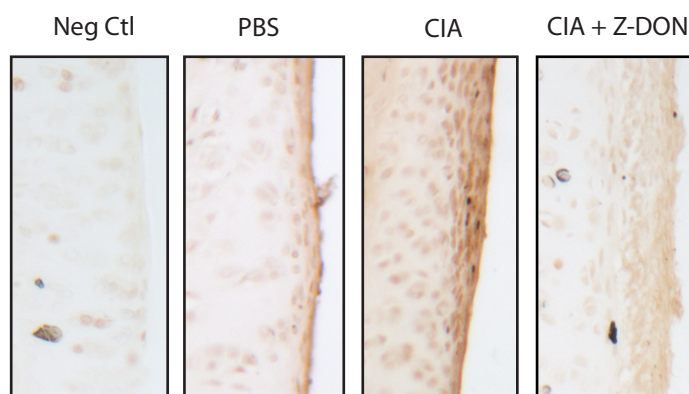

Supplement: Additional file 2 — Figure S2. TG2 is linked to the cross-linking activity in synovial tissues. (A) Synovial tissues from control (PBS) and CIA rats were incubated with biotin-pentylamide (PAB) in the presence or absence of 250 μM KCC-009 (KCC) or 100 μM Z-DON and revealed by Western blotting. (B) PAB was injected in PBS or CIA articulations with or without Z-DON (100 μM) and revealed by immunochemistry with streptavidin-peroxidase. Neg Ctl, control tissue without biotin-pentylamide. [file ar3899-S2.PDF]

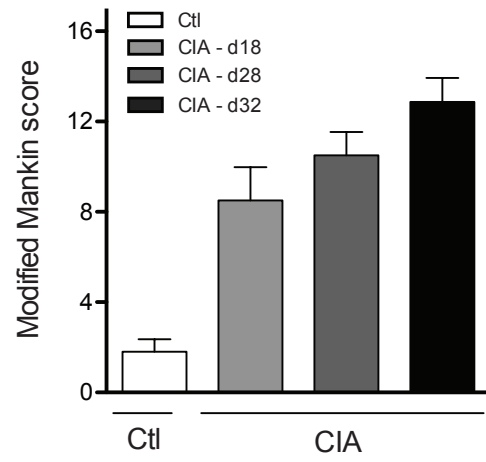

Supplement: Additional file 3 — Figure S3. Evaluation of CIA progression by using a modified Mankin scoring system. Histopathologic classification of the severity of arthritic lesions was evaluated by using a modified Mankin scoring system. Graph shows mean values ± SEM for control and arthritic rats at different times (18, 28, and 32 days) after immunization with type II collagen (Ctl, n = 4; CIA, n = 8). [file ar3899-S3.PDF]

A

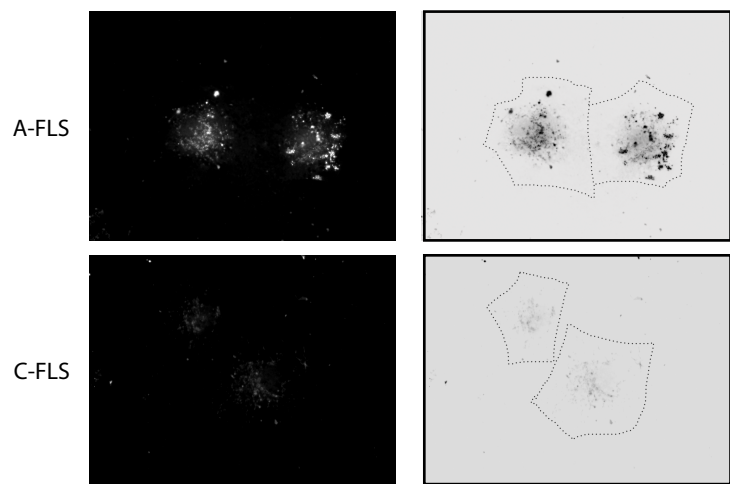

B

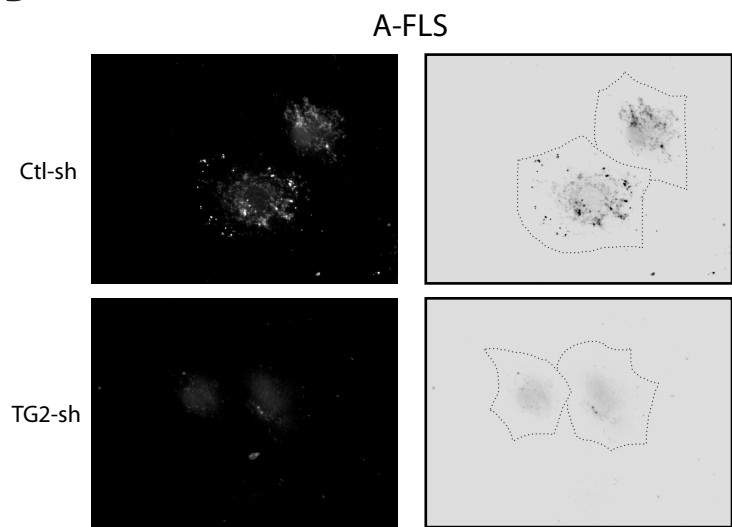

C

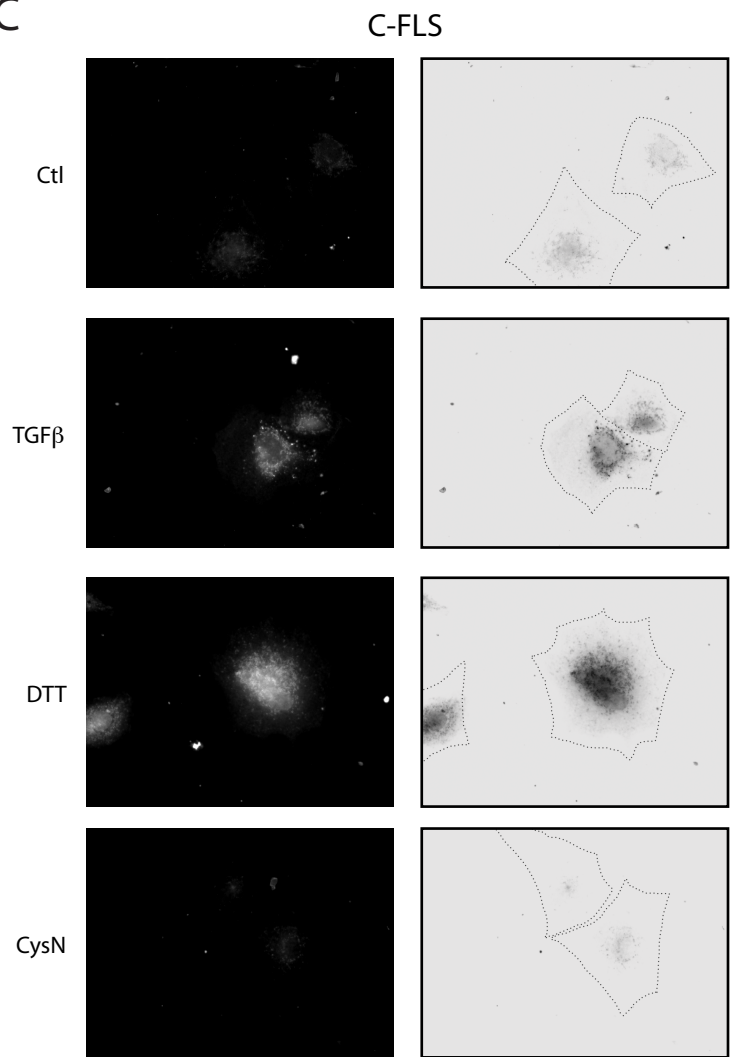

Supplement: Additional file 4 — Figure S4. Modulation of TGase activity is associated with TG2 regulation. (A-C) TGase activity was measured by using in situ 5-(biotinamido)-pentylamine incorporation assay in (A) C-FLSs or A-FLSs cultured on gelatin matrix. (B) A-FLSs were transfected with control- or TG2-shRNA-expressing lentivirus. (C) C-FLSs were incubated with TGF-β (20 ng/ml), DTT (1 mM), or cystamine (100 μΜ). [file ar3899-S4.PDF]

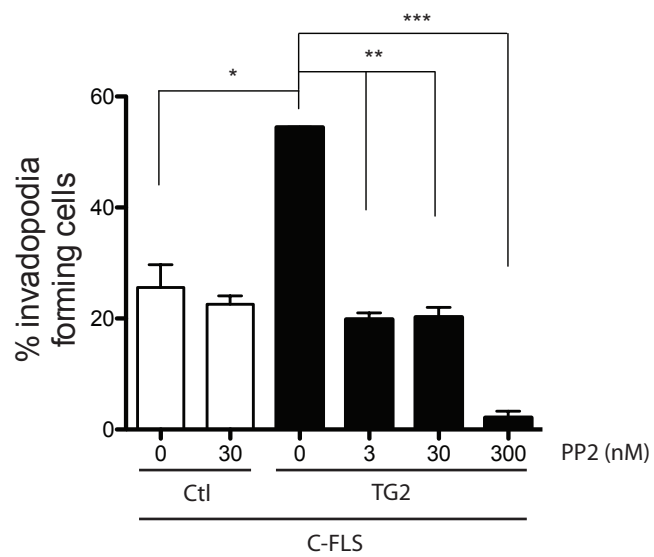

Supplement: Additional file 5 — Figure S5. Src is implicated in TG2-mediated invadopodia formation. C-FLSs were transfected with a TG2-expressing or a control (empty) plasmid and cultured on gelatin in the presence or absence of PP2. After 24 hours, the percentage of invadopodia-forming cells was counted. Values are expressed as the mean ± SEM (*P < 0.05; **P < 0.01; ***P < 0.001). [file ar3899-S5.PDF]
